# Vaccine Virus

**Published:** 1854-04

**Authors:** 


Vaccine Virus.—Our medical friends are informed that we have now a supply of fresh vaccine virus. It is our intention henceforth to be able to meet all demands upon us for this article. The sum of one dollar accompanying the order will be promptly attended to, if prepaid. This small fee will aid in the liquidation of the expenses of our Journal, to which object it will be applied.

